# Development of a Lozenge for Oral Transmucosal Delivery of *Trans*-Resveratrol in Humans: Proof of Concept

**DOI:** 10.1371/journal.pone.0090131

**Published:** 2014-02-26

**Authors:** Otis L. Blanchard, Gregory Friesenhahn, Martin A. Javors, James M. Smoliga

**Affiliations:** 1 Wilmore Labs, LLC, San Antonio, Texas, United States of America; 2 University of Texas Health Science Center at San Antonio, Psychiatry, San Antonio, Texas, United States of America; 3 Department of Physical Therapy, High Point University, High Point, North Carolina, United States of America; National Cancer Institute at Frederick, United States of America

## Abstract

Resveratrol provides multiple physiologic benefits which promote healthspan in various model species and clinical trials support continued exploration of resveratrol treatment in humans. However, there remains concern regarding low bioavailability and wide inter-individual differences in absorption and metabolism in humans, which suggests a great need to develop novel methods for resveratrol delivery. We hypothesized that oral transmucosal delivery, using a lozenge composed of a resveratrol-excipient matrix, would allow resveratrol to be absorbed rapidly into the bloodstream. We pursued proof of concept through two experiments. In the first experiment, the solubility of *trans*-resveratrol (*t*RES) in water and 2.0 M solutions of dextrose, fructose, ribose, sucrose, and xylitol was determined using HPLC. Independent *t*-tests with a Bonferroni correction were used to compare the solubility of *t*RES in each of the solutions to that in water. *t*RES was significantly more soluble in the ribose solution (p = 0.0013) than in the other four solutions. Given the enhanced solubility of *t*RES in a ribose solution, a resveratrol-ribose matrix was developed into a lozenge suitable for human consumption. Lozenges were prepared, each containing 146±5.5 mg *t*RES per 2000 mg of lozenge mass. Two healthy human participants consumed one of the prepared lozenges following an overnight fast. Venipuncture was performed immediately before and 15, 30, 45, and 60 minutes following lozenge administration. Maximal plasma concentrations (*C*_max_) for *t*RES alone (i.e., resveratrol metabolites not included) were 325 and 332 ng⋅mL^−1^ for the two participants at 15 minute post-administration for both individuals. These results suggest a resveratrol-ribose matrix lozenge can achieve greater *C*_max_ and enter the bloodstream faster than previously reported dosage forms for gastrointestinal absorption. While this study is limited by small sample size and only one method of resveratrol delivery, it does provide proof of concept to support further exploration of novel delivery methods for resveratrol administration.

## Introduction

Resveratrol is a stilbene polyphenol found in grapes and other plant products which has received considerable attention in the past decade for its potential to improve health and combat age-related diseases [1]. A considerable number of *in vitro* and animal model studies have revealed *trans*-resveratrol (*t*RES) has a plethora of potential beneficial effects, including prevention of atherosclerosis, improving glucose tolerance, enhancing endurance capacity, and reducing inflammation [1], [2]. Full discussions of physiological effects and their mechanisms have been reviewed elsewhere and are beyond the scope of this paper. Multiple published papers have described the results of *t*RES treatment in humans, and most research suggests that *t*RES is safe and could indeed improve cardiometabolic health, especially in individuals with chronic disease [3].

One of the chief limitations in developing mainstream clinical applications for *t*RES is overcoming the limited bioavailability to consistently achieve concentrations which have been demonstrated to be effective in laboratory models, and there is a need to improve bioavailability of *t*RES [4]. The dose and concentration of *t*RES in blood in human trials are often below the doses used in animal and cell culture studies [5]–[8], even with multiple daily oral doses [9]. Although some studies have shown beneficial effects following low-dose *t*RES treatment in clinical populations [10], there is evidence that some of the beneficial physiologic effects of *t*RES treatment require large dosages [11]. However, high oral dosages may result in mild to severe side effects, depending on the patient population. For instance, at doses higher than 2,500 mg, *t*RES has been shown to cause gastrointestinal upset and diarrhea in healthy individuals [11] and exacerbate gastrointestinal ulcers in laboratory models [12]. Additionally, there is concern regarding high doses of *t*RES in certain clinical populations. A 5,000 mg daily dose of a proprietary *t*RES formulation has been associated with renal failure in multiple myeloma patients [13], where existing renal dysfunction may have been exacerbated by a large quantity of resveratrol metabolites. These side effects have limited the oral dose in most human trials to 1,000 mg or lower [14]. In fact, most commercially available supplements contain ≤500 mg *t*RES [15].

Human bioavailability studies utilizing gastrointestinal absorption of resveratrol (i.e., ingestion of powders, capsules, caplets, etc.) have found wide inter-individual variability in *t*RES absorption, plasma concentration of free *t*RES, and resveratrol metabolite profile [5], [6], [8], [9]. This is in part due to extensive metabolism of *t*RES. First-pass metabolism, the modification of drug during initial absorbance in the small intestine and liver, greatly limits the plasma concentrations of *t*RES attained following absorption of a standard oral dose [6], [8], [16]. It has long been known that *t*RES is extensively glycosolated and sulfalated during absorption in the small intestine [17]–[20]. Recent evidence suggests that gut microbiota may also play a role in metabolism of *t*RES before it reaches the bloodstream, contributing to inter-individual variability in bioavailability and metabolite profile [21]. Like other molecules absorbed through the intestinal tract, resveratrol is then transported to the liver, where it is further metabolized before it enters the systemic circulation [17], [18], [22]. Indeed, it is possible that unmodified *t*RES may demonstrate significant physiological effects once it reaches the plasma, but the magnitude of these effects may be limited if absorption and metabolism limit plasma concentration and bioavailability. Thus, oral transmucosal (OTM) delivery, which circumvents the gut and first-pass hepatic metabolism may increase the absorption of *t*RES and substantially reduce inter-individual variability in peak plasma concentration and metabolite profile to allow for greater clinical utility.

An OTM formulation such as a lozenge could circumvent the physiological factors limiting absorption and bioavailability of *t*RES which normally occur following administration of a pill, food, or liquid [5], [23], [24]. Resveratrol has mixed potential as a candidate for OTM absorption. Favorable properties include relatively small molecular weight of *t*RES (228 g⋅mol^−1^). Additionally, *t*RES is uncharged at physiological pH which will allow passive diffusion across the buccal mucosa [25], [26]. This is experimentally supported where *t*RES has been shown to have high flux in cultured oral epithelial tissue [27]. The aqueous solubility is a limiting factor in exploring OTM dosing, where transcellular rate of diffusion is based on the concentration of drug in solution or free form. The other limiting factor for OTM formulations of *t*RES is dose size, which is restricted to approximately 100–150 mg for a lozenge [25]. This may be sufficient to achieve a physiologic effect if absorption is sufficiently high. Other OTM delivery forms, such as films and sprays are limited to a much smaller dose size.

We hypothesized that incorporation of *t*RES into a simple lozenge would allow its adequate absorption through the oral mucosa of humans. Thus, the first purpose of our study was to determine if the aqueous solubility of *t*RES would be affected by combining it with selected excipients to optimize the likelihood of significant OTM absorption. The second purpose of the study was to determine the maximal plasma concentration (C_max_) of *t*RES following administration of a *t*RES lozenge to healthy human volunteers. The results of these initial experiments provide proof-of-concept for the development of a *t*RES lozenge using appropriate excipients, which could lead to greater exploration of novel delivery methods for *t*RES administration and ultimately lead to new clinical applications.

## Methods

This study included two components: 1) a high performance liquid chromatography (HPLC) analysis of *t*RES solubility in various concentrations of different sugar/sugar alcohol excipients, and 2) a human pilot study to determine the peak plasma concentration of *t*RES achieved following administration of an optimized *t*RES lozenge.

### Solubility of *Trans*-resveratrol

Stock *t*RES solution was made with a ratio of 5 mg of *t*RES dissolved in 0.50 mL of methanol. Directly after making stock solution, 12 µL of *t*RES/Methanol was transferred to a 2 mL microcentrifuge tube. The methanol was allowed to evaporate completely (approximately 6 hours) while shielded from light and loosely covered with aluminum foil leaving 12 µg of dry *t*RES in the tube. This was qualitatively confirmed visually by the remaining yellowish residue, consistent with that of dry resveratrol. Dry tubes were stored overnight at 4°C and allowed to warm to room temperature before addition of 2.0 M solutions or dH20 to measure *t*RES solubility.

Twenty milliliter 2.0 M solutions of dextrose, fructose, ribose, sucrose, and xylitol were prepared using measured quantities of dry reagents purchased from Sigma Aldrich and de-ionized water. Solutions were mixed over low heat until fully dissolved the evening before the study and stored at 4°C in glass vials. The respective stock solutions were transferred to the prepared microcentrifuge tubes containing dry *t*RES and diluted with de-ionized water in an appropriate ratio for a final concentration of sugar or polyalcohol of 2 M and a volume of 1 ml. The tubes were sealed, vortexed, and stored protected from light for approximately 18 hours at room temperature.

To measure aqueous *t*RES concentrations, each sample was first centrifuged for 3 minutes at 16.1 k×g to pellet excess *t*RES, leaving all dissolved *t*RES in the supernatant. Extra care was taken not to disturb the loose, off white/yellow pellet. To prepare sample for HPLC, 200 µl of each sample was transferred to a fresh tube diluted with 400 µl methanol, and then filtered for injection on the chromatography column.

Chromatography was performed on a C18 column (250 mm×4.6 mm, 3 µm). *Trans-*resveratrol was detected by UV at 214 nm and eluted at 3∶00 min. Running buffer was 20 mM phosphate buffer (6.8 pH):methanol at a 25%:75% ratio. A volume of 150 µL of each prepared sample was analyzed by HPLC. This method was adapted from Singh et al [28]. Solubility of *t*RES was measured in at least triplicate for water and each of the other solutions.

#### Statistical analysis

Mean and standard deviation for *t*RES solubility in water and each of the solutions were computed. Independent t-tests were used to compare the solubility of *t*RES between water, and each of the excipient solutions. Statistical significance was set *a priori* with α = 0.050. A Bonferroni correction was then applied to reduce Type I error, and given the three comparisons, p≤0.010 was considered statistically significant (0.050/5 = 0.010).

### Bioavailability of Resveratrol Lozenge with Ribose to Improve Aqueous Solubility

#### Ethics statement

All procedures relating to human experimentation in the human bioavailability study were approved by the Institutional Review Board at High Point University. Human participants underwent written informed consent.

#### Lozenge preparation and content verification

Based on the results of the first experiment, ribose was chosen as an excipient to enhance solubility of *t*RES in a lozenge. The preparation of a 2000 mg lozenge containing approximately 46% ribose, 46% (fructose/sucrose mixture), and 8% *t*RES was adapted from hard lozenge techniques from using published recommendations from a university pharmaceutics and compounding laboratory [29] and traditional jewel candy recipes [30]. *Trans-*resveratrol was purchased from Biotivia Longevity Pharmaceuticals, LLC (New York, NY, USA). All ingredients measured from dry weight. Lozenges were stored in lozenge molds wrapped in aluminum foil and a sealed plastic bag at −20°C.

Final concentration of *t*RES in lozenges were measured by the Biological Psychiatry Analytical Labs at The University of Texas at San Antonio. A 1 mg sample was collected from random prepared lozenges and the concentration of *t*RES was quantified for each lozenge using the HPLC methods described previously. The mass of the remaining lozenge was then measured and multiplied by the *t*RES concentration to compute the total *t*RES dose for each lozenge.

#### Participants

Individuals at least 18 years of age with no history of cardiorespiratory, neurological, or metabolic disease were eligible to participate in this pilot study. Individuals regularly taking any type of medication or a recent history of supplements containing material derived from grapes (e.g., resveratrol, grape seed extract) or other polyphenols were excluded from the study.

#### Lozenge administration procedures

Participants arrived to the laboratory in a fasted state, such that they did not ingest anything besides water since the previous midnight. After undergoing informed consent, a fasting blood sample was obtained from the antecubital vein. Participants were provided the *t*RES-ribose lozenge and verbally instructed how to ingest it. Participants were told to keep the lozenge placed between their gum and cheek until it was completely dissolved and minimize swallowing any of their saliva or pieces of the lozenge.

The time at which the lozenge was initially administered was recorded. A venous blood sample was then obtained every fifteen minutes thereafter for one hour. Only one hour of data were obtained because oral trans-mucosal absorption would be expected to be rapid and occur within this time frame [25]. All blood samples were collected into EDTA tubes, which were then placed in an ice bath before being centrifuged to separate red blood cells from plasma. The retained plasma was stored at −80°C until the time of analysis (<2 weeks).

Participants were asked to report any unusual or adverse events.

#### Peak plasma concentration analysis

Resveratrol was quantified in plasma using HPLC with UV detection. Briefly, 200 µL of calibrators and unknown samples were mixed with 10 µL of 100 µg/mL flurbiprofen (internal standard) and 2 mL of 50∶50 acetonitrile:methanol. The samples were vortexed vigorously and then centrifuged at 1500 *g* for 15 min. Supernatants were transferred to glass test tubes and dried to residue under a gentle stream of nitrogen. The residues were redissolved in 250 µL of mobile phase and then filtered using a microfilterfuge tube. Then, 150 µL of the calibrators, controls, and final samples were injected into the HPLC. The ratios of the peak area of resveratrol to that of the internal standard flurbiprofen were compared against a linear regression of calibrators at concentrations of 0, 50, 100, 500, and 1000 ng/ml to quantify resveratrol in the samples. Resveratrol concentration in plasma was reported in ng/mL.

The HPLC system consisted of an Alltima C18 column (4.6×150 mm, 5 µm), Waters 2487 UV detector, Waters 717 autosampler, Waters 515 HPLC pump. The mobile phase was 35% acetonitrile, 64.9% Milli-Q water, and 0.1% phosphoric acid (pH 2.5). The flow rate of the mobile phase was 1.5 mL/min and the wavelength of absorbance was 214 nm.

#### Statistical analysis

Given that *C*_max_ data were only obtained from two human subjects using only one treatment (lozenge comparing *t*RES-ribose matrix), data are reported from each subject and no other formal statistical comparisons were possible.

## Results

### Solubility of *Trans*-resveratrol

Solubility of *t*RES in water and across the 2.0M concentrations of the dextrose, fructose, ribose, sucrose, and xylitol solutions are presented in **TABLE 1**. The solubility of *t*RES was significantly enhanced in the 2.0M ribose solution (p = 0.0013), but not in fructose (p = 0.0950), dextrose (p = 0.2284), sucrose (p = 0.5824), or xylitol (p = 0.3330).

**Table 1:** Solubility of resveratrol in different aqueous solutions (Mean ± Standard Deviation).

Solution	Water (n = 5)	2.0M Dextrose(n = 4)	2.0M Fructose(n = 4)	2.0M Ribose(n = 3)	2.0M Sucrose(n = 3)	2.0M Xylitol(n = 3)
**Solubility**	2.02±0.33	1.57±0.68	2.41±0.25	3.60±0.47*	1.80±0.80	2.30±0.41

Values are in arbitrary units derived from HPLC.

* = Statistically significant from water at the p≤0.010 (Bonferroni correction) level.

### Lozenges

Four lozenges were successfully created using the basic techniques described in the methods. The *t*RES content of each is displayed in **TABLE 2**, with a mean ± SD of 146.2±5.5 mg *t*RES per 2000 mg lozenge mass.

**Table 2:** Trans-resveratrol content of four individual lozenges created.

ID #	Lozenge [*t*RES] (µg/mg)	Estimated *t*RES dose per 2000 mg lozenge (mg)
Ribose Loz A1	74.9	149.8
Ribose Loz A2	72.1	144.2
Ribose Loz B1	69.7	139.4
Ribose Loz B2	75.7	151.4

### Bioavailability of Optimized Lozenge

#### Human participants

Two healthy male individuals received the *t*RES-ribose matrix lozenge and had their plasma *C*_max_ for *t*RES quantified. A summary of the participant data is present in **TABLE 3**. Both participants reported the lozenge had a mild pepper-like taste and that they were able to follow the administration procedures properly. Neither participant reported noticing anything unusual or any adverse events following the optimized *t*RES lozenge administration.

**Table 3:** Data from human participants (both male).

	Participant 1	Participant 2
**Age (y)**	32	34
**Height (m)**	1.70	1.78
**Body Mass (kg)**	60.2	75.7
***t*** **RES ** ***C*** **_max_ (ng**⋅**mL** ^−**1**^ **)**	325	332

Note that the maximal plasma concentration (*C*_max_) is that for trans-resveratrol (*t*RES) alone and therefore does not include any resveratrol metabolites.

#### Peak plasma concentration and time to maximal concentration

**Figure 1:**
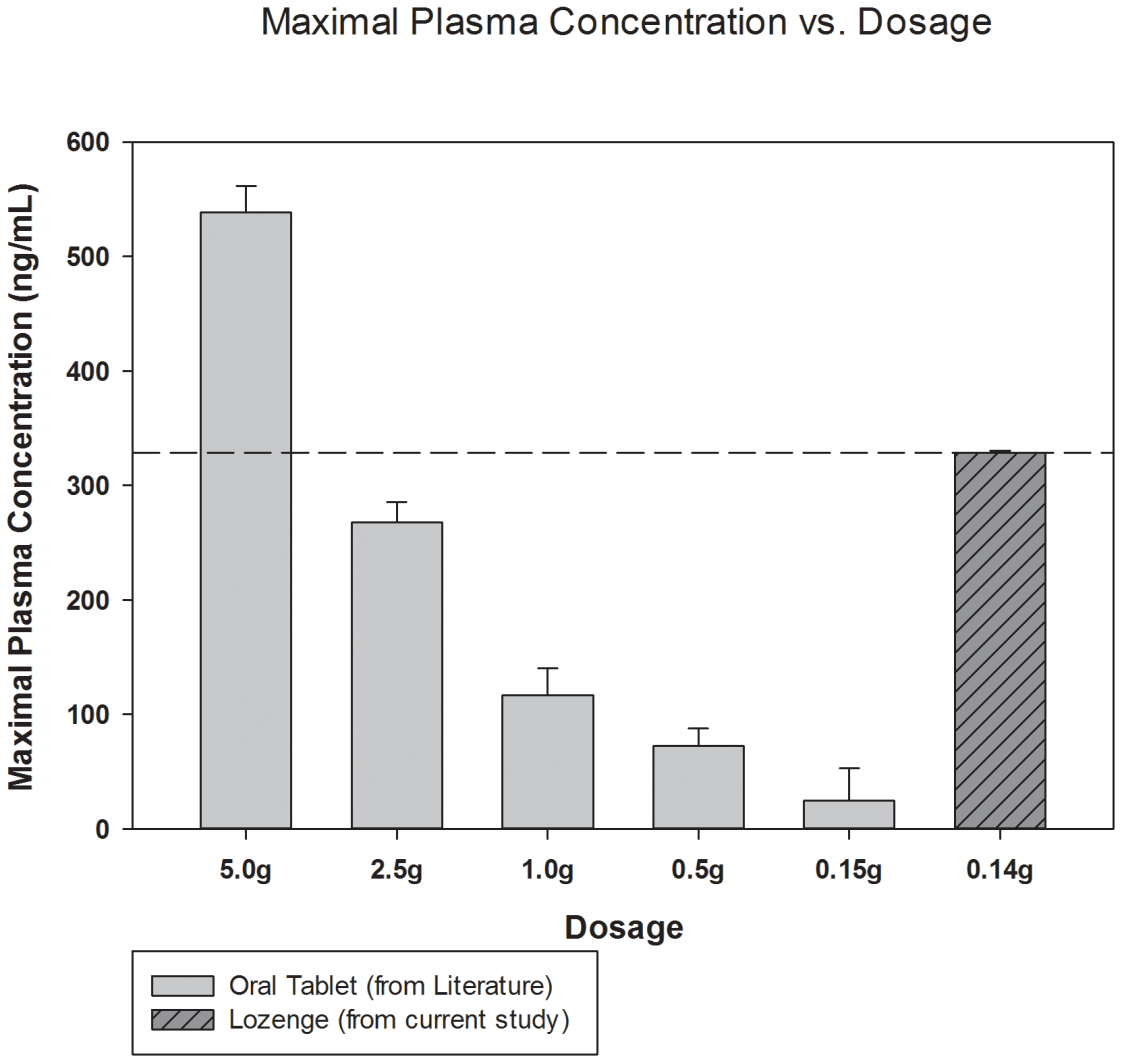
Peak plasma concentration (*C*_max_) of *t*RES following administration of a lozenge in reference to previously reported data. Note that the maximal plasma concentration (*C*_max_) is that for trans-resveratrol (*t*RES) alone and therefore does not include any resveratrol metabolites.

**Figure 2:**
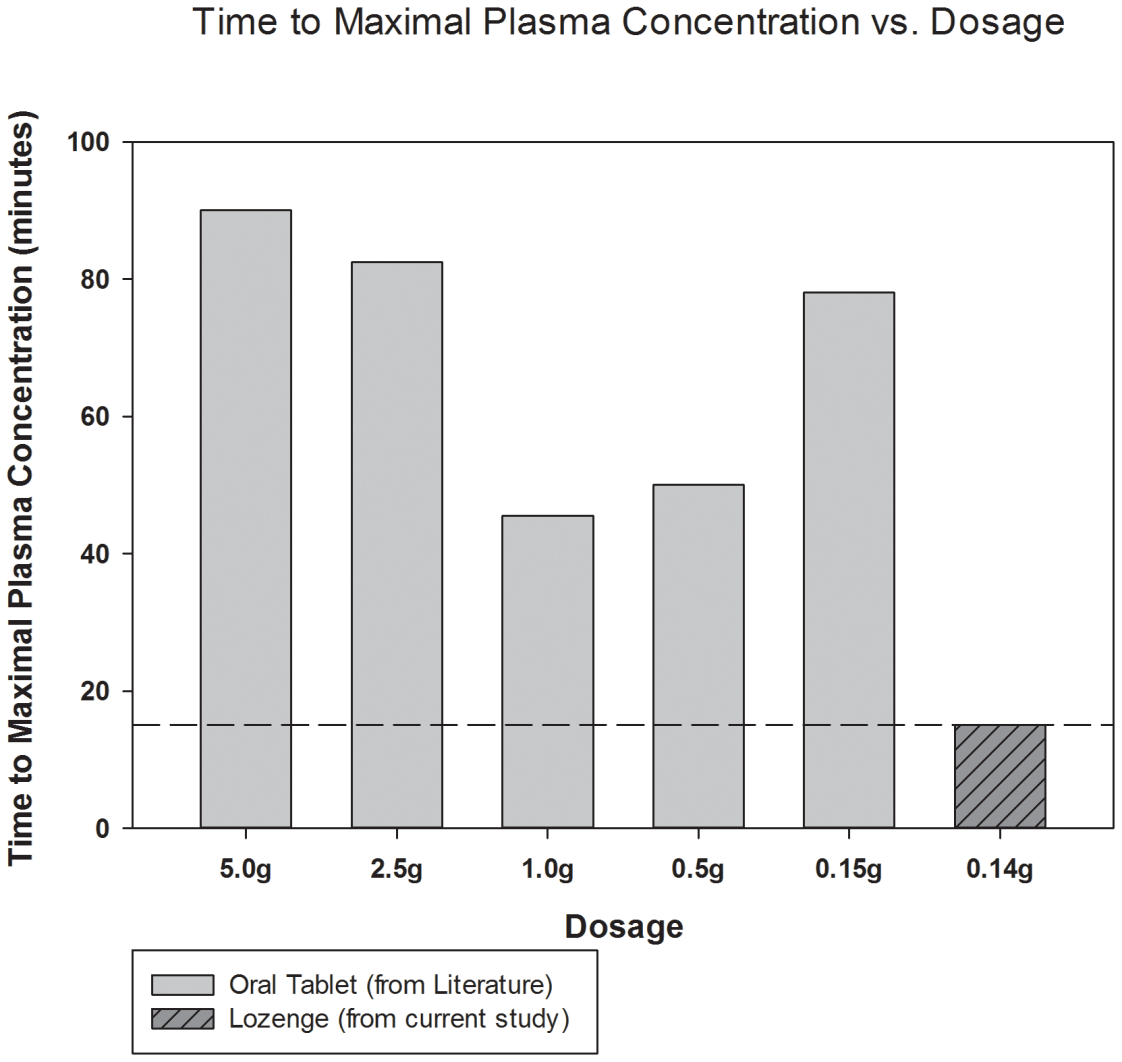
Time to peak plasma concentration of *t*RES following administration of a lozenge in reference to previously reported data.

At baseline, plasma concentration of *t*RES was below detectable limits and therefore presumed to be zero. Plasma concentrations peaked 15 minutes following lozenge administration (Participant 1: *C*_max_ = 325 ng⋅mL^−1^; Participant 2: *C*_max_ = 332 ng⋅mL^−1^). *Trans*-resveratrol concentrations were below detectable limits at the 30, 45, and 60 post-administration time points. A comparison of these data in reference to previously reported data are presented in **FIGURE 1** and **FIGURE 2**.

## Discussion

The results of this study demonstrate that the aqueous solubility of *t*RES can be enhanced using ribose, and that OTM absorption of *t*RES using a lozenge is possible. The OTM delivery method appears to elevate peak plasma level of *t*RES above that previously reported following GI absorption (e.g., powder, caplets) in humans. Given the limited number of excipients tested, limited dosage forms, and the small number of human participants this should be considered a proof of concept study which provides the first evidence to support further pursuit towards development of a resveratrol lozenge optimized for human OTM absorption.

### Solubility of *Trans*-resveratrol

The first aim of our study was to determine if the aqueous solubility of *t*RES would be affected by dextrose, fructose, ribose, sucrose, or xylitol. Major factors determining a successful drug candidate for OTM dosing is amount of drug which is in solution in the aqueous environment of the mouth with absorption being due to passive diffusion [25], [31]. As stated by Zhang et al “The effective formulation must not only release the drug to the mucosal surface, but do so with the drug in its free form.” Any compound which increases resveratrol’s aqueous solubility and form a strong complex with resveratrol, such as is possible with detergents or cyclodextrins, could prevent or slow absorption and we’re not considered for this study [25]. With the low aqueous solubility *t*RES, any change in the aqueous solubility of resveratrol may have a significant impact on the pharmacokinetics of an OTM dose.

It is difficult to predict the effect of sugars as cosolvents on the aqueous solubility of solutes, such as *t*RES, where increases or decreases with differing concentration may occur with little clear explanation [32]. Sugars are understood to create order within an aqueous solution, and that there can be a “sugaring out” effect on aromatic organic molecules [32]–[34]. If an excipient, such as sucrose, were to decrease the aqueous solubility of *t*RES, its inclusion in an OTM dosage would slow absorption across the oral mucosa. This would reduce the concentration of free *t*RES in solution in the oral cavity, reduce the rate of flux of tRES across the mucous membrane and increase the amounts being swallowed and undergoing the less efficient gastrointestinal absorption. However, there were no statistically significant observed effects on the aqueous solubility *t*RES solubility for the tested excipients such as dextrose, fructose, sucrose, and xylitol (**TABLE 1** **)**.

Sugars may also increase the solubility for poorly soluble aromatic organic molecules such as biphenyl, hexane, naphthalene, and adenine [35], [36]. Ribose was shown to be the strongest solubolizer of the aforementioned hydrocarbons [35], and we discovered that 2.0M ribose did significantly increase the aqueous solubility of *t*RES (**TABLE 1** **)**. The α-D-ribopyranose isomer of ribose has all hydroxyl group on a single face, leaving the opposing face largely non-polar [35], [37]. This dual-faced nature of the pyranose form of ribose has been credited to enhance biphenyl’s and benzene’s aqueous solubility [35], [38], as well as enhance ribose’s permeation across lipid bilayers [37]. The increase in aqueous solubility of *t*RES by ribose may be due to this same mechanism. Future research may explore the use of other excipients, alone or in combination, to determine if the aqueous solubility of resveratrol can be further increased, and the impact of increased solubility on OTM dosing.

A number of other studies have attempted to increase the oral bioavailability of resveratrol by combining it with other related molecules. For instance, piperine (a polyphenol found in black pepper) has been found to improve the GI bioavailability of resveratrol in mice [39]. Likewise, there has been success in substantially increasing bioavailability by improving solubility of resveratrol, as recently demonstrated through the use of highly soluble GI caplets, though this is complicated by greater inter-individual variability compared to standard *t*RES powder [4]. While these matrices do increase plasma bioavailability compared to resveratrol powder and may cause synergistic or additive effects, they still are subject to the limitations of gastrointestinal absorption, including metabolism, dose size from, wide inter-individual variability in absorption, and GI side effects.

### Oral Transmucosal Absorption of *Trans*-resveratrol in Humans

With limited resources, we focused on developing only one lozenge formulation, using a matrix of approximately 140 mg resveratrol with ribose. In the two participants tested, lozenge administration resulted in a C_max_ of free unmodified *t*RES of 328.5±5.0 ng⋅mL^−1^ at 15 minutes after administration of the OTM lozenge, which is a considerably higher *C*_max_ than that reported from administration of a similar or greater oral dosages utilizing gastrointestinal absorption (e.g. [6], [9], FIGURE 1). For example, Nunes et al [40] tested the pharmacokinetics for a single 200 mg oral dose in humans and the mean *C*_max_ was measured to be approximately 25 ng⋅ml^−1^ at 0.8 hours. A dosage of 500 ng⋅ml^−1^ by Brown et al [11], the *C*_max_ of unalterated resveratrol was 43.8 ng/ml at one hour. The primary contributing factor to the increased unmodified *t*RES *C*_max_ compared those previously reported for standard oral dosing is likely the avoidance of GI tract, which allowed the relatively low dose of *t*RES in the lozenge to be sufficiently absorbed into the bloodstream via the oral mucous membrane without first being glycosolated and sulfalated in the intestines followed by first pass hepatic metabolism [8].

Peak plasma concentration was observed in both of the participants fifteen minutes following lozenge administration, which is substantially faster than reported using traditional oral supplements using gastrointestinal absorption (FIGURE 2). This is likely due elimination of the time necessary for a capsule to travel to the intestines and for the contents of a capsule to be released and absorbed. Since blood samples were collected at fifteen minute intervals, it is possible that the actual peaks may have been achieved slightly earlier or slightly later (yet before the 30 minute sample). Nonetheless, this is considerably quicker than time to peak concentration reported following that of a traditional oral *t*RES supplement [6], [9] or a highly soluble caplet [4]. However, it must be noted that plasma free *t*RES concentration was not measurable at the 30 minute time point. Thus, it was not possible to compute the area under the curve (AUC) for *t*RES, and therefore the total *t*RES exposure could not be determined and compared to that reported previously.

Given this was a proof of concept study rather than a full-scale bioavailability study, resveratrol metabolites were not measured. It remains unknown whether OTM absorption influences metabolite profile. It remains unclear how biologically relevant these metabolites are in humans, but there is evidence that these metabolites have physiologic effects in laboratory models [41]–[44]. Therefore, it is too early to determine whether it is advantageous, deleterious, or inconsequential to avoid metabolism of *t*RES in the gut. However, given previous reports of limited absorption, OTM delivery does provide a potential avenue to maximize absorption, thereby making *t*RES administration more cost effective, and also reduce inter-individual variability in plasma concentration. Additionally, the lozenge may be advantageous in reducing the risk of gastrointestinal side effects.

The concept of using a lozenge to deliver a therapeutic compound via OTM absorption is well established [25], [31], [45], yet the pursuit of this delivery method has been largely neglected for *t*RES administration. Notable therapeutic advances are rooted in implementation of OTM dosage when oral dosages of the same drug were less effective [25], [31]. For instance, sublingual tablets of nitroglycerine are used to provide rapid relief from angina by increasing cardiovascular blood flow, and have been in use for over 100 years [46]. Likewise, various formulations for OTM delivery of opioid analgesic drugs (e.g., fentanyl citrate) have provided a significant advantage in pain management over their oral counterparts [47]. Further, this is not the first report to suggest an OTM administration for resveratrol [48]. Asensi et al [49] briefly report that a 50 ml solution of unspecified fluid containing ∼1 mg of resveratrol retained in the mouth for 60 seconds and achieved maximum plasma concentration comparable to a 250 mg oral dose in humans, though further details of the methods and results are not provided.

### Future Applications of Oral Transmucosal Delivery of *Trans*-resveratrol

It remains unknown whether the benefits of *t*RES are dependent on intermittent peaks or sustained elevation in plasma *t*RES concentration, given that both exposure time to *t*RES and concentration influences cellular response [50]. Recent evidence from cancer cells and animal models suggests that resveratrol metabolites may be enzymatically converted back into parent compound (“recycling”) which may allow for a sustained release of trans-resveratrol [51]. However, it is not known whether this occurs in all types of tissue (e.g., healthy and cancerous human breast tissue does not have measurable levels of SULT1A1 [52], which plays a major role in resveratrol metabolism [22]). We hypothesize that the rapid clearance of free *t*RES resulted from metabolism of the parent compound, such that the later samples likely contained high concentrations of resveratrol metabolites. Indeed, these metabolites could potentially be recycled back into parent compound, similar to that from standard oral dosages.

Although limited, our results provide some encouraging data to suggest that OTM administration may allow for more rapid absorption of *t*RES, which may have strong clinical applications. Regardless of absorption into other tissues, endothelial cells would receive rapid bolus of *t*RES. This suggests there is potential for *t*RES to be used in medical management of ischemic events (e.g., angina), given its ability to acutely improve blood flow and vascular function in humans [53], [54] and protect from or attenuate ischemic injuries in laboratory models [55]–[57]. Likewise, a rapidly deliverable form of *t*RES may be beneficial in treating acute trauma to counter the development of oxidative stress and inflammation (e.g., concussion or spinal cord injury), which has also been supported through animal models [58]–[61]. It must be stated that these concepts of using rapid-delivery of *t*RES are based on sound theory which are supported with our bioavailability data from two human participants, but further validation of these data is required before initiating human clinical trials in this realm.

### Study Limitations

Although our data is novel and may have implications in the development of future *t*RES products, we acknowledge a number of limitations to this study. First and foremost, the limited number of human participants and solubility measurements requires that measurements must be considered proof of concept, rather than a full bioavailability study. While ribose was found to be the excipient which resulted in the greatest solubility, this is not necessarily the optimal formulation. Further research must be conducted to determine if different molecules, alone or in combination, can further enhance the solubility of resveratrol. While it is clear that ribose increases aqueous solubility of *t*RES, it is not yet certain whether this actually enhances OTM absorption. As such, future studies should compare lozenges composed of different resveratrol matrices. Further, only free *t*RES plasma concentration was measured, and the plasma metabolite profile was not measured. Thus, future research exploring OTM absorption of resveratrol should investigate whether the bioavailability of *t*RES is truly greater compared to standard or other novel delivery methods. Future studies should also include a dose curve, as 140 mg is a very large OTM dose and may not be the most efficient dosage. Lastly, these data demonstrate high peak plasma concentrations achieved very quickly, but do not provide information about total resveratrol absorption, as measured by area under the curve. Nonetheless, the data provides a proof-of-concept that it is possible to reach plasma levels which have elicited physiological effects through OTM dosage form of resveratrol.

## Conclusions

In summary, the results of this study demonstrate that ribose increases the aqueous solubility of *t*RES compared to its solubility in water alone. Further, OTM administration of a lozenge containing a mixture of ribose with resveratrol results in a very high peak plasma concentration compared to that reported from similar dosages of free resveratrol administered as a traditional oral supplement. Likewise, peak plasma concentration of free resveratrol was achieved approximately 15 minutes following lozenge administration, which is considerably quicker than the 60–120 minutes reported using traditional free resveratrol tablets. As human clinical trials investigate the benefits of *t*RES treatment on human health, it will become especially important to develop effective delivery methods which are cost effective, produce minimal side effects, and limit inter-individual bioavailability. Future research must be conducted to determine if the high plasma levels attained from the optimized resveratrol lozenge hold true across multiple individuals and whether the metabolite profile differs considerably from that obtained from an oral supplement.
